# Effects of regular spectacle wear on binocular accommodative function in myopic adults with dry eye disease

**DOI:** 10.3389/fmed.2025.1717444

**Published:** 2025-12-15

**Authors:** Yingpin Cao, Dan Sun, Yi Liang, Jiajia Ye, Bin Wu, Ming-ming Yang

**Affiliations:** 1 Department of Ophthalmology, Shenzhen People’s Hospital (The First Affiliated Hospital, Southern University of Science and Technology, The Second Clinical Medical College, Jinan University), Shenzhen, Guangdong, China; 2 Hunan Provincial University Key Laboratory of the Fundamental and Clinical Research on Neurodegenerative Diseases, Changsha Medical University, Changsha, Hunan, China

**Keywords:** binocular accommodative function, dry eye disease, myopia, spectacle correction, tear film stability

## Abstract

**Objective:**

To evaluate the impact of regular refractive correction (spectacle wear) on dry-eye-related parameters and binocular visual function in myopic adults with coexisting dry eye disease (DED).

**Methods:**

A cross-sectional observational study enrolled 120 myopic patients with DED, divided into a regular spectacle-wear group (*n* = 68) and a minimal/non-wear group (*n* = 52) based on daily spectacle wear duration. Dry eye parameters including tear meniscus height (TMH) and average tear break-up time (Ave TBUT), binocular accommodative function parameters including positive and negative relative accommodation (PRA/NRA), monocular and binocular accommodative facility (AF), and Ocular Surface Disease Index (OSDI) scores were assessed. Correlations between Ave TBUT, TMH, and binocular accommodative function were analysed.

**Results:**

The regular spectacle-wear group showed significantly longer Ave TBUT and lower OSDI scores (*p* < 0.05), with no significant TMH difference. Binocular accommodative function was better in the regular spectacle wear group, with significantly lower PRA, higher NRA, and greater monocular/binocular AF (all *p* < 0.05). Ave TBUT was negatively correlated with PRA and positively correlated with NRA and AF (*p* < 0.05); TMH showed no significant correlations.

**Conclusion:**

Regular spectacle wear is associated with improved tear film stability, reduced dry-eye symptoms, and enhanced binocular visual function in myopic patients with dry eye.

## 1 Introduction

Myopia is the most prevalent refractive error globally, and its prevention, control, and visual health management represent a critical public health issue (1). According to World Health Organization projections, the global myopic population will reach 4.76 billion by 2050. Among these individuals, those with high myopia face a significantly increased risk of vision-threatening fundus pathologies such as macular degeneration and retinal detachment (2). Spectacles (glasses), being the most common optical correction for myopia (3), yet the long-term effects of spectacle wear on ocular surface homeostasis and binocular visual function have not been comprehensively investigated. While recent research has focused on the adverse impacts of contact lenses (e.g., orthokeratology lenses) on ocular surface inflammatory factors (such as IL-17A) and tear film stability, high-quality studies examining the association between this fundamental correction method (spectacles) and ocular surface health remain scarce (4–7). In particular, systematic evaluation of core tear film stability parameters (e.g., TMH and TBUT) and their association with ocular surface symptoms is lacking.

Current research suggests an indirect association between myopia correction methods and the development or progression of DED (8, 9). The undercorrection of myopia has been demonstrated to induce persistent accommodative lag and convergence dysfunction (10–12), thereby exacerbating asthenopic symptoms—a recognized significant trigger for DED (13, 14). Notably, research investigating the correlation between binocular accommodative parameters and refractive correction status, particularly with spectacles, remains insufficient. Previous studies have been hampered by significant limitations: current research in this field is limited by several methodological shortcomings. Most studies adopt a unidimensional approach, restricting analyses to either ocular surface parameters or binocular accommodative function parameters independently (15–18). Integrative investigations that simultaneously assess and correlate ocular surface health with binocular accommodative function indicators remain scarce. Furthermore, many studies inadequately control for key confounding factors, including daily lens wear duration, intensity of near work, and screen time exposure, which may substantially influence outcomes.

Building upon these research gaps and the potential underlying mechanisms, this study hypothesises that regular wear of fully corrected spectacles may contribute to improved tear film stability and more balanced accommodation–convergence function, potentially alleviating dry eye symptoms. We propose that this effect arise, at least in part, from enhanced retinal image quality and reduced binocular accommodative function anomalies, such as persistent accommodative lag induced by blurred imagery. To test this hypothesis, we employed the standardised dry eye diagnostic protocol recommended by the Tear Film and Ocular Surface Society Dry Eye Workshop II (TFOS DEWS II) (19, 20), combined with a comprehensive binocular accommodative function assessment. The aim is to systematically compare differences in ocular surface health status (including objective metrics such as TMH and TBUT, as well as subjective OSDI scores) and binocular accommodative function between two cohorts within a population of individuals with both myopia and DED: those consistently wearing fully corrected spectacles versus those without regular refractive correction. By integrating ocular surface and binocular accommodative function assessments, this investigation seeks to provide more targeted clinical evidence to optimise myopia correction strategies for this specific patient population.

## 2 Methods

### 2.1 General information

Adult patients (aged 18–45 years) with myopia and coexisting DED, diagnosed and treated at the Ophthalmology Clinic of Shenzhen People’s Hospital between January 2023 and January 2025, were screened. Based on the inclusion and exclusion criteria, 120 participants were ultimately enrolled. This study included two comparison groups within the same clinical population (myopic adults with DED) according to their average daily spectacle wear duration: a regular spectacle-wear group (*n* = 68 patients; 136 eyes) and a minimal/non-wear group (*n* = 52 patients; 104 eyes) served as the behavioral control group. This cross-sectional observational study was approved by the Ethics Review Committee of Shenzhen People’s Hospital. The study strictly adhered to the principles of the Declaration of Helsinki. Written informed consent was obtained from all participants prior to enrollment.

### 2.2 Inclusion and exclusion criteria

#### 2.2.1 Inclusion criteria

Participants were required to have a best-corrected visual acuity (BCVA) of 1.0 or better in both eyes. All participants had been diagnosed with myopia in both eyes for more than 1 year, with a spherical equivalent (SE) refractive error between −6.00 D and −0.50 D (inclusive) in each eye. The interocular difference in spherical power was ≤1.50 D, and the interocular difference in cylindrical power was ≤1.00 D. Daily electronic screen use time was ≤8 h.

- Regular spectacle-wear group: Refractive correction was achieved exclusively with spectacles fitted with non-functional lenses (e.g., without blue-light blocking coatings). The difference between the spectacle lens prescription and the actual refractive error determined at the study visit was ≤±0.50 D. Spectacles were worn for ≥12 h per day.
- Minimal/non-wear group: Participants had been diagnosed with myopia but either had never undergone refractive correction (spectacle wear) or had not received sustained, effective refractive correction (we defined as cumulative spectacle wear duration ≤2 h per day).

#### 2.2.2 Exclusion criteria

Participants were excluded if they had a history of autoimmune diseases, used topical ocular medications within the past month, had active ocular surface inflammation, presented with strabismus, had organic ocular diseases (e.g., cataracts, glaucoma), had vitamin D or vitamin A deficiency, had chronic systemic diseases such as thyroid disorders, hypertension or diabetes mellitus, were contact lens wearers, had a history of ocular surgery or corneal disease, had chronic occupational/environmental exposure to dust, chemical irritants, constant high-wind conditions or prolonged exposure to low-humidity air conditioning; or were unable to cooperate with the required examinations.

### 2.3 Diagnostic criteria

DED was diagnosed in this study according to the evidence-based guidelines outlined in the Second Report of the International Dry Eye Workshop (TFOS DEWS II, 2017). The diagnosis of DED required the concurrent fulfillment of both of the following criteria.

#### 2.3.1 Subjective symptoms

- Presence of persistent ocular surface discomfort symptoms (e.g., dryness, burning sensation, foreign body sensation, eye fatigue or fluctuating vision).
- A total score ≥13 (range: 0–100) on the standardised OSDI questionnaire.

#### 2.3.2 Objective signs (at least one of the following)

Abnormal tear film stability: Mean tear film breakup time (TFBUT) ≤5 s (average of three consecutive measurements), assessed via either non-invasive break-up time (NIBUT) or fluorescein break-up time (FBUT).
Ocular surface damage: Positive corneal fluorescein staining (≥Grade 1 according to the Oxford Staining Score grading system, range: 0–5).
Participants meeting both the symptom criteria and at least one sign criterion were diagnosed with DED.

### 2.4 Refraction assessment

All participants underwent refractive evaluations at their initial visit, performed by the same optometrist. Objective refraction was conducted using an automatic refractometer (model: TOPCON KR-2000, Topcon, Japan) to obtain preliminary refractive data, including spherical power, cylindrical power and axis. This was followed by subjective refraction in a semi-dark room using a phoropter to determine the final refractive prescription.

### 2.5 Dry eye evaluation

All dry-eye-related examinations were performed in a dedicated, environmentally controlled room (ambient temperature: 68 °F–77 °F; relative humidity: 40%RH–60%RH). Prior to testing, participants were instructed to sit quietly in the examination room for at least 10 min to acclimate to the environmental conditions, allowing the tear film to stabilise. All procedures were conducted by a single experienced technician who had received rigorous training in standardised operating procedures (SOPs). The examination sequence followed a predefined protocol to minimize cross-interference amongst tests.

NIBUT and TMH were measured using a comprehensive dry eye analyser (Keratograph^®^ 5M, OCULUS Optikgeräte GmbH, Germany). The device was calibrated and operated strictly according to the manufacturer’s instructions before and during use.

### 2.6 Measurement principles and parameters

#### 2.6.1 NIBUT

The device utilises a corneal topography system based on Placido ring projection to continuously capture tear film images. Built-in software algorithms automatically analyse the dynamic process of tear film break-up. Three consecutive measurements were taken, and the average value (in seconds) was recorded.

#### 2.6.2 TMH

The device employs infrared imaging technology to automatically detect the lower lid margin and measure the tear meniscus. The TMH value was recorded in micrometers (μm).

#### 2.6.3 Binocular accommodative function assessment

Binocular accommodative function tests were performed after full refractive correction in all participants. The evaluation included PRA, NRA and AF.

##### 2.6.3.1 PRA/NRA

Using the near vision chart on the phoropter at a testing distance of 40 cm, participants were instructed to fixate on a 20/30 optotype under their corrected distance prescription.

NRA was assessed first by incrementally adding +0.25 diopter (D) plus lenses until the participant reported sustained blurring of the target. The maximum plus lens power added before the sustained blur was recorded as the NRA value.

PRA was subsequently measured by adding −0.25 D minus lenses under the same conditions until the participant reported sustained blur. The maximum minus lens power added was recorded as the PRA value.

##### 2.6.3.2 AF

AF was measured at 40 cm using a single 20/30 optotype. Monocular testing was performed first, followed by binocular testing.

##### 2.6.3.3 Monocular AF

One eye was occluded, while the other was presented with alternating ±2.00 D lenses using flippers. The participant was instructed to refocus and clarify the optotype with each lens change. One complete cycle of positive and negative lens clearing was counted as one cycle per minute (cpm). The number of cycles completed in one minute was recorded.

##### 2.6.3.4 Binocular AF

After removing the occluder, the participant repeated the procedure binocularly, and the binocular AF (cpm) was documented.

#### 2.6.4 OSDI questionnaire

All participants completed the 12-item OSDI questionnaire, designed to evaluate symptoms related to DED and their impact on vision-related functioning. The questionnaire comprises three subscales: ocular symptoms, vision-related functions and environmental triggers. Each item was rated on a scale from 0 to 4 (0 = none of the time; 1 = some of the time; 2 = half of the time; 3 = most of the time; 4 = all of the time). The final score was calculated using the following formula:


(Sumofallscores×25)÷Number of questions answered,

yielding a total score ranging from 0 to 100. OSDI scores were interpreted as follows: 0–12 = normal, 13–22 = mild dry eye, 23–32 = moderate dry eye, 33–100 = severe dry eye.

#### 2.6.5 Visual behaviors questionnaire assessment

Information on visual behaviors was obtained using a structured questionnaire administered at the study visit. Participants were asked to report their average daily screen time (hours/day, including computer, tablet, and smartphone use), average near work time (hours/day, including reading, writing, and screen activities at ≤50 cm), and their usual near-working distance (cm, self-reported as the typical distance when reading or using a digital device). Each item was collected as a continuous variable and cross-checked for plausibility. For analysis, values were expressed as mean ± SD and minimum–maximum range.

In addition to symptom scoring and visual behaviors, the questionnaire included demographic information such as gender, age, estimated daily duration of spectacle wear.

### 2.7 Masking

All dry-eye-related and binocular vision assessments were performed by a trained examiner who was masked to group allocation and spectacle-wear history. Participants were instructed not to disclose their wear habits during testing. The objective measurements (TBUT, TMH, PRA, NRA, AF) followed standardised protocols and were not influenced by examiner judgment.

### 2.8 Statistical analysis

Data were analysed using IBM SPSS Statistics version 25. Categorical variables were presented as frequencies (percentages). Continuous variables were described based on their distribution: Normally distributed data were expressed as mean ± standard deviation (SD), while non-normally distributed data were presented based on the median (minimum, maximum).

Between-group comparisons were conducted as follows: chi-square tests were used for categorical variables; independent samples *t*-tests were applied to normally distributed continuous variables; and non-normally distributed continuous variables were compared using the Mann–Whitney *U* test.

For correlation analysis, Pearson correlation coefficients were used for normally distributed variables, while Spearman-rank correlation coefficients were applied for variables not following a normal distribution. Based on normality tests (e.g., the Shapiro–Wilk test), all dry eye parameters, binocular vision function metrics and questionnaire scores were found to be non-normally distributed. Therefore, the Mann–Whitney *U* test was used for all relevant group comparisons. Participants were stratified into four severity categories—normal, mild, moderate, and severe dry eye—according to OSDI score criteria. As these were ordinal variables, intergroup comparisons were also conducted using the rank-sum test. Given the non-normal distribution of both dry eye indices and binocular accommodative function parameters, their correlations were assessed using Spearman rank correlation analysis.

## 3 Results

### 3.1 Patient demographics

A total of 120 subjects were included in this study, including 35 males (29. 1%) and 85 females (70.8%), aged 21 to 45 years, with an average age of 32.7 years. They were divided into a regular spectacle-wear group and a minimal/non-wear group based on the duration with which they wore frame glasses every day. All participants fulfilled TFOS DEWS II diagnostic criteria, including symptoms (OSDI ≥13) and at least one objective sign (TBUT ≤5 s or positive corneal staining). The basic information about the two groups is detailed in Table 1.

**Table 1:** Basic information of the regular spectacle-wear group and the minimal/non-wear group (*n* = 120).

Baseline characteristics	The regular spectacle-wear group	The minimal/non-wear group	Statistic	*p*-value
Gender, *n* (%)			0.005^a^	0.946
Male	20 (29.4)	15 (28.8)		
Female	48 (70.6)	37 (71.2)		
Age, mean ± SD	31.43 ± 10.15	33.85 ± 9.76	−1.323^b^	0.189
SE (OD), median (min, max)	−2.00 (−4.50, −0.50)	−2.00 (−4.25, −0.50)	−0.298^c^	0.766
SE (OS), median (min, max)	−2.25 (−5.50, −0.25)	−2.00 (−3.75, −0.25)	−0.893^c^	0.372
Average daily screen time (h/day, mean ± SD)	7.6 ± 2.5 (3–12)	7.4 ± 2.3 (3–11)	−0.45^b^	0.653
Near work time (h/day, mean ± SD)	5.2 ± 2.1 (2–10)	5.0 ± 2.0 (2–9)	−0.47^b^	0.641
Usual near-working distance [cm, median (min, max)]	32 (25–45)	33 (25–46)	−0.39^c^	0.694

SE, spherical equivalent.

a *χ*^2^-value.

b *t*-value.

c *Z*-value.

### 3.2 Comparison of dry eye parameters between the two groups

The rank-sum test showed no significant difference in TMH between the groups. However, a statistically significant difference was observed in average TBUT, with both eyes in the regular spectacle-wear group demonstrating longer average TBUT values than those in the minimal/non-wear group (see Table 2).

**Table 2:** Comparison of dry eye parameters between the regular spectacle-wear group and minimal/non-wear group (*n* = 120).

Parameters related to dry eye examination	The regular spectacle-wear group median (min, max)	The minimal/non-wear group median (min, max)	*Z*-value	*p*-value
TMH (mm)				
OD	0.15 (0.04, 0.48)	0.15 (0.05, 0.38)	1.346	0.179
OS	0.14 (0.04, 0.27)	0.15 (0.05, 0.39)	1.428	0.185
Ave TBUT(s)				
OD	6.25 (2.20, 12.00)	1.50 (0.50, 5.60)	−8.754	<0.001
OS	6.00 (2.00, 14.00)	1.50 (0.50, 5.00)	−8.933	<0.001

TMH, tear meniscus height; Ave TBUT, average tear break-up time.

### 3.3 Comparison of binocular accommodative parameters between the two groups

The rank-sum test revealed statistically significant differences across all parameters. The regular spectacle-wear group demonstrated lower PRA but higher NRA, monocular AF for both eyes and binocular AF compared to the minimal/non-wear group (see Table 3).

**Table 3:** Comparison of binocular accommodative function parameters between the regular spectacle-wear group and the minimal/non-wear group (*n* = 120).

Accommodative function parameters	The regular spectacle-wear group median (min, max)	The minimal/non-wear group median (min, max)	*Z*-value	*p*-value
PRA	−3.00 (−5.00, −0.75)	−1.00 (−3.50, 0.00)	−7.066	<0.001
NRA	2.50 (1.25, 3.75)	0.75 (0.00, 3.50)	−7.673	<0.001
AF (cpm)				
OD	11 (0.5, 15)	3 (0, 14)	−6.686	<0.001
OS	11 (0.5, 15)	2.5 (0, 13)	−6.064	<0.001
OU AF (cpm)	8 (0, 13)	2 (0, 12)	−5.877	<0.001

PRA, positive relative accommodation; NRA, negative relative accommodation; AF, accommodative facility; cpm, cycle per minute.

### 3.4 Comparison of OSDI scores between groups

A statistically significant difference in OSDI scores was observed between the two groups. The refractive correction group demonstrated significantly lower OSDI scores—indicating less severe dry eye symptoms—compared to the minimal/non-wear group (see Table 4).

**Table 4:** Comparison of dry eye questionnaire scores between the regular spectacle wear group and the minimal/non-wear correction group (*n* = 120).

Questionnaire scores	The regular spectacle-wear group	The minimal/non-wear group	*Z*-value	*p*-value
OSDI questionnaire score, median (min, max)	24 (9, 54)	39.5 (11, 66)	−5.003	<0.001
OSDI dry eye severity classification, *n* (%)				
Normal	2 (2.9)	1 (1.9)	−4.835	<0.001
Mild dry eye	26 (38.2)	8 (15.4)		
Moderate dry eye	31 (45.6)	11 (21.2)		
Severe dry eye	9 (13.2)	32 (61.5)		

OSDI, ocular surface disease index.

### 3.5 Correlation analysis between dry eye parameters and binocular accommodative function

No statistically significant differences were found between the left and right eyes in terms ofTMH, Ave TBUT or monocular AF (*p* = 0.976, 0.535 and 0.968, respectively). Thus, right-eye dry eye parameters (TMH, Ave TBUT) were analysed for correlations with binocular accommodative parameters. TMH showed no significant correlations with any binocular vision parameters in either group. However, Ave TBUT demonstrated significant correlations with all binocular accommodative parameters (*p* < 0.05), exhibiting a negative correlation with PRA but positive correlations with NRA, monocular AF and binocular AF (see Table 5).

**Table 5:** Analysis of correlation between right-eye dry eye metrics and binocular accommodative function parameters (*n* = 120).

Binocular visual function parameters	TMH	Ave TBUT		
	Spearman correlation coefficient	*p*-value	Spearman correlation coefficient	*p*-value
PRA	0.111	0.228	−0.521	<0.001
NRA	−0.179	0.051	0.588	<0.001
OD AF (cpm)	0.016	0.867	0.429	<0.001
OU AF (cpm)	0.024	0.798	0.464	<0.001

TMH, tear meniscus height; Ave TBUT, average tear break-up time; PRA, positive relative accommodation; NRA, negative relative accommodation; AF, accommodative facility; cpm, cycle per minute.

## 4 Discussion

This study investigated the association between regular optical refractive correction (spectacle wear) and dry eye-related parameters and binocular visual function in patients with myopia complicated by DED. The results demonstrated that the average TBUT was significantly longer in the regular spectacle-wear group compared to the minimal/non-wear group, while TMH showed no statistically significant difference between groups. TMH, defined as the vertical height of the tear meniscus at the lower lid margin, serves as an objective parameter to evaluate tear volume and to assist in dry eye subtyping (21). One of the primary determinants of TMH is the volume of tear secretion (22), which includes basal secretion—mainly produced by accessory lacrimal glands (Krause and Wolfring glands) under autonomic nervous control—and reflex secretion—primarily derived from the main lacrimal gland in response to ocular surface stimuli such as dryness or foreign body sensation. There is no established evidence supporting a direct correlation between spectacle wear and basal tear production. Moreover, regular spectacle wear does not typically activate or suppress the reflex tear secretion pathway, which is stimulus-dependent. Given the relative independence between tear secretion volume and the mode of refractive correction (in this study, conventional spectacle lenses), the use of spectacles is unlikely to significantly affect TMH in patients with myopia and DED. Therefore, the lack of a significant difference in TMH between groups aligns with the current physiological understanding of tear production. TBUT, which measures the interval (in seconds) between a complete blink and the initial appearance of tear film breakup, is a key indicator of tear film stability (23). Spectacles may help reduce tear evaporation by shielding the ocular surface from direct exposure to airflow or environmental desiccating factors such as wind or air conditioning (24). Previous studies have confirmed that increased tear evaporation is a central contributor to dry eye pathogenesis (25–27). Uncorrected myopic patients frequently squint or widen their palpebral fissure to compensate for blurred vision, these behaviors may accelerate tear evaporation. Improved visual quality following refractive correction may reduce these compensatory actions, contributing to more stable tear film dynamics. Our findings suggest that spectacle wear in myopic individuals is associated with better tear film stability compared to uncorrected counterparts. Although there is limited direct evidence that single-vision spectacles themselves alleviate dry eye symptoms, the existing literature suggests that progressive addition lenses (PALs) may help mitigate visual fatigue and dry eye symptoms associated with visual display terminal (VDT) use (28). It’s well established that VDT work can induce or exacerbate evaporative dry eye through mechanisms such as decreased blink rate, increased incomplete blinking and enhanced tear film evaporation (29, 30). Taken together, these findings support the hypothesis that refractive correction through spectacles may indirectly support tear film stability by reducing visual strain and mitigating factors that contribute to ocular surface stress. This may provide novel clinical insight into the management of patients with coexisting myopia and dry eye, suggesting that spectacles could be a preferred refractive correction option relative to contact lenses for myopic patients with dry eye symptoms.

Our study also revealed that, compared to the uncorrected group, the regular spectacle-wear group exhibited lower PRA values but higher NRA values and both monocular and binocular AF values. These findings suggest that refractive correction is associated with significant differences in binocular visual function, particularly accommodation. PRA reflects the capacity of the accommodative system to exert accommodative effort. A decrease in PRA after spectacle correction may be attributed to improved retinal image quality and reduced accommodative demand. Enhanced NRA and AF values in the corrected group may indicate a more stable accommodative system post-correction, facilitating both accommodative relaxation and dynamic efficiency. In contrast, uncorrected myopic individuals, due to persistent blurred retinal images, may develop abnormal accommodative responses characterised by increased accommodative effort, manifesting as higher PRA, lower NRA and reduced AF—hallmarks of accommodative excess and relaxation insufficiency. These findings underscore the importance of clear retinal images in maintaining normal accommodative function, likely mediated by improved neural feedback following image clarity, which enhances the coordination between accommodative stimuli and responses (31).

A significant correlation was observed between average TBUT and binocular accommodative function, particularly accommodative parameters. Tear film instability may impair accommodative amplitude and reduce AF by degrading corneal optical quality, disrupting neural feedback and inducing visual fatigue. Tear film stability is a fundamental prerequisite for maintaining optical clarity and high-quality visual input (32). TBUT, as a critical indicator of tear film stability, is associated with tear film dynamics; its reduction may increase anterior corneal surface irregularities, leading to elevated higher-order aberrations (HOAs) and decreased contrast sensitivity on the retina (33, 34). Clinically, this manifests as blurred vision, glare and other visual disturbances, thereby reducing image quality. Previous studies have demonstrated that patients with TBUT <5 s demonstrate significantly reduced contrast sensitivity across all spatial frequencies, likely due to increased corneal light scatter and decreased modulation transfer function (MTF) (35, 36). In our study, TBUT was significantly negatively correlated with PRA (*r* = −0.521, *p* < 0.001). This may be explained by the compensatory accommodative effort triggered by persistent blurred retinal images due to tear film instability, which may reduce accommodative reserves over time. Furthermore, elevated HOAs and reduced MTF may impair the accommodative system’s responsiveness to defocus stimuli.

TBUT was also positively correlated with NRA (*r* = 0.588, *p* < 0.001), suggesting that tear film stability supports high-quality retinal image formation and reduces abnormal accommodative stimulation. A stable tear film may help maintain an intact corneal–trigeminal–parasympathetic reflex arc, thereby preventing accommodative spasm. Additionally, TBUT exhibited positive correlations with both monocular AF (*r* = 0.429) and binocular AF (*r* = 0.464, *p* < 0.001), possibly reflecting reduced optical fluctuations during lens switching and improved ciliary muscle–iris sphincter coordination supported by stable tear film and intact neural conduction. This study highlights the importance of evaluating binocular visual function in patients with DED in clinical practice. In addition to conventional ocular surface assessments such as TBUT and Schirmer’s test, it is recommended that binocular accommodative function tests, particularly accommodative function (e.g., AF, NRA, PRA etc.), should be conducted in myopic DED patients who are uncorrected or who present with symptoms of visual fatigue. For DED patients with concomitant accommodative dysfunction, a combined therapeutic approach involving both targeted dry eye treatment and binocular vision therapy may improve visual quality and alleviate ocular discomfort.

To our knowledge, few studies have systematically examined the combined relationship between ocular surface parameters and binocular visual function in myopic patients with dry eye. Our findings suggest that regular spectacle correction may be associated with more stable tear film dynamics and accommodative function. While this raises the possibility that spectacles could be a preferable correction option for patients with coexisting myopia and dry eye, prospective studies are required to confirm these associations before clinical recommendations can be made. Given the cross-sectional observational design of this study, residual behavioral bias cannot be fully excluded. Nevertheless, the use of standardised measurement procedures and masked outcome assessment helped minimize measurement-related bias. Accordingly, the findings should be interpreted as associations rather than evidence of causality. Future studies should adopt prospective interventional designs to longitudinally monitor changes in dry eye signs, symptoms and accommodative function in previously uncorrected myopic patients with DED following standardised spectacle correction. This would allow the further validation of the causal effects of refractive correction on the optimisation of binocular accommodative function and the maintenance of tear film homeostasis. The study did not include a healthy control group without DED, which limits generalizability to non-DED populations. Future prospective studies could incorporate a normal control group to further validate the findings. Such evidence would support the precise refinement of clinical management strategies and promote the establishment of an integrated model for the co-management of refractive error and dry eye.

## Data Availability

The raw data supporting the conclusions of this article will be made available by the authors, without undue reservation.

## References

[1] Sankaridurg P Tahhan N Kandel H Naduvilath T Zou H Frick KD . IMI impact of myopia. Invest Ophthalmol Vis Sci. (2021) 62:2. doi: 10.1167/iovs.62.5.2, 33909036 PMC8083082

[2] Holden BA Fricke TR Wilson DA Jong M Naidoo KS Sankaridurg P . Global prevalence of myopia and high myopia and temporal trends from 2000 through 2050. Ophthalmology. (2016) 123:1036–42. doi: 10.1016/j.ophtha.2016.01.006, 26875007

[3] Hashemi H Fotouhi A Yekta A Pakzad R Ostadimoghaddam H Khabazkhoob M. Global and regional estimates of prevalence of refractive errors: systematic review and meta-analysis. J Curr Ophthalmol. (2017) 30:3–22. doi: 10.1016/j.joco.2017.08.009, 29564404 PMC5859285

[4] Stapleton F Alves M Bunya VY Jalbert I Lekhanont K Malet F . TFOS DEWS II epidemiology report. Ocul Surf. (2017) 15:334–65. doi: 10.1016/j.jtos.2017.05.003, 28736337

[5] Aćimović L Stanojlović S Kalezić T Dačić Krnjaja B. Evaluation of dry eye symptoms and risk factors among medical students in Serbia. PLoS One. (2022) 17:e0275624. doi: 10.1371/journal.pone.0275624, 36279260 PMC9591051

[6] Uchino M Dogru M Uchino Y Fukagawa K Shimmura S Takebayashi T . Japan Ministry of Health study on prevalence of dry eye disease among Japanese high school students. Am J Ophthalmol. (2008) 146:925–929.e2. doi: 10.1016/j.ajo.2008.06.030, 18723141

[7] Zhang Y Chen H Wu X. Prevalence and risk factors associated with dry eye syndrome among senior high school students in a county of Shandong Province, China. Ophthalmic Epidemiol. (2012) 19:226–30. doi: 10.3109/09286586.2012.670742, 22650150

[8] Shen Z Shi K Yu Y Yu X Lin Y Yao K. Small incision lenticule extraction (SMILE) versus femtosecond laser-assisted *in situ* keratomileusis (FS-LASIK) for myopia: a systematic review and Meta-analysis. PLoS One. (2016) 11:e0158176. doi: 10.1371/journal.pone.0158176, 27367803 PMC4930219

[9] Qian L Wei W. Identified risk factors for dry eye syndrome: a systematic review and meta-analysis. PLoS One. (2022) 17:e0271267. doi: 10.1371/journal.pone.0271267, 35984830 PMC9390932

[10] Logan NS Radhakrishnan H Cruickshank FE Allen PM Bandela PK Davies LN . IMI accommodation and binocular vision in myopia development and progression. Invest Ophthalmol Vis Sci. (2021) 62:4. doi: 10.1167/iovs.62.5.4, 33909034 PMC8083074

[11] Li SM Li SY Liu LR Guo JY Chen W Wang NL . Full correction and undercorrection of myopia evaluation trial: design and baseline data of a randomized, controlled, double-blind trial. Clin Experiment Ophthalmol. (2013) 41:329–38. doi: 10.1111/j.1442-9071.2012.02884.x, 23009037

[12] Ong ACH Cruickshank FE Sheppard AL Davies LN. The efficacy of multifocal soft contact lenses for the alleviation of asthenopic symptoms in myopes with accommodative lag. Cont Lens Anterior Eye. (2022) 45:101514. doi: 10.1016/j.clae.2021.101514, 34511307

[13] Sheppard AL Wolffsohn JS. Digital eye strain: prevalence, measurement and amelioration. BMJ Open Ophthalmol. (2018) 3:e000146. doi: 10.1136/bmjophth-2018-000146, 29963645 PMC6020759

[14] Auffret É Gomart G Bourcier T Gaucher D Speeg-Schatz C Sauer A. Digital eye strain. Symptoms, prevalence, pathophysiology, and management. J Fr Ophtalmol. (2021) 44:1605–10. doi: 10.1016/j.jfo.2020.10.002 34657757

[15] Kumar NR Praveen M Narasimhan R Khamar P D’Souza S Sinha-Roy A . Tear biomarkers in dry eye disease: progress in the last decade. Indian J Ophthalmol. (2023) 71:1190–202. doi: 10.4103/IJO.IJO_2981_22 37026250 PMC10276712

[16] Li J Liao Y Zhang SY Jin L Congdon N Fan Z . Effect of laughter exercise versus 0.1% sodium hyaluronic acid on ocular surface discomfort in dry eye disease: non-inferiority randomised controlled trial. BMJ. (2024) 386:e080474. doi: 10.1136/bmj-2024-080474, 39260878 PMC12036614

[17] Yoon HJ Moon HS Sung MS Park SW Heo H. Effects of prolonged use of virtual reality smartphone-based head-mounted display on visual parameters: a randomised controlled trial. Sci Rep. (2021) 11:15382. doi: 10.1038/s41598-021-94680-w, 34321504 PMC8319184

[18] Hsiao CJ Tung HC Tien CL Chang YW Cheng CY. The influence of large-diameter multifocal contact lens on ocular surface, visual quality, and binocular accommodative function for presbyopic adults with dry eye syndromes. Sci Rep. (2023) 13:19444. doi: 10.1038/s41598-023-46732-6, 37945680 PMC10636056

[19] Berta A Tóth-Molnár E Csutak A. New international consensus statement about the definition, classification, ethiology, diagnostics and therapy of dry eye (TFOS DEWS II). Orv Hetil. (2018) 159:775–85. doi: 10.1556/650.2018.31077 29754511

[20] Wang MTM Power B Xue AL Kim JS Craig JP. Diagnostic performance of qualitative and quantitative methods of meibomian gland dropout evaluation in dry eye disease: an investigator-masked, randomised crossover study. Cont Lens Anterior Eye. (2025) 48:102324. doi: 10.1016/j.clae.2024.102324 39557589

[21] Craig JP Nichols KK Akpek EK Caffery B Dua HS Joo CK . TFOS DEWS II definition and classification report. Ocul Surf. (2017) 15:276–83. doi: 10.1016/j.jtos.2017.05.008, 28736335

[22] Li J Shen M Wang J Ma H Tao A Xu S . Clinical significance of tear menisci in dry eye. Eye Contact Lens. (2012) 38:183–7. doi: 10.1097/ICL.0b013e318252ce0c 22543731

[23] Papas E. Tear break-up time: clinical procedures and their effects. Ophthalmic Physiol Opt. (1999) 19:274–5. doi: 10.1046/j.1475-1313.1999.00422.x, 10627847

[24] Park KS Lee BJ Ang MJ Eslani M Shacterman S Jun J . Investigating mask-associated dry eye and contributing factors in healthcare providers. Clin Ophthalmol. (2025) 19:1443–54. doi: 10.2147/OPTH.S510917, 40330002 PMC12050417

[25] Sheppard J Shen Lee B Periman LM. Dry eye disease: identification and therapeutic strategies for primary care clinicians and clinical specialists. Ann Med. (2023) 55:241–52. doi: 10.1080/07853890.2022.2157477, 36576348 PMC9809411

[26] Karpecki PM Nichols KK Sheppard JD. Addressing excessive evaporation: an unmet need in dry eye disease. Am J Manag Care. (2023) 29:239–47. doi: 10.37765/ajmc.2023.89448 37844320

[27] Monfared N Murphy PJ. Features and influences on the normal tear evaporation rate. Cont Lens Anterior Eye. (2023) 46:101809. doi: 10.1016/j.clae.2022.101809, 36621341

[28] Alionis A Netto A Netto T Alves M. Evaluation of the effects of single vision lenses with additional near-power on computer-induced asthenopia. Rev Bras Oftalmol. (2020) 79:325–9. doi: 10.5935/0034-7280.20200069

[29] Akkaya S Atakan T Acikalin B Aksoy S Ozkurt Y. Effects of long-term computer use on eye dryness. North Clin Istanb. (2018) 5:319–22. doi: 10.14744/nci.2017.54036, 30859162 PMC6371992

[30] Kamøy B Magno M Nøland ST Moe MC Petrovski G Vehof J . Video display terminal use and dry eye: preventive measures and future perspectives. Acta Ophthalmol. (2022) 100:723–39. doi: 10.1111/aos.15105, 35122403 PMC9790652

[31] Read JCA Kaspiris-Rousellis C Wood TS Wu B Vlaskamp BNS Schor CM. Seeing the future: predictive control in neural models of ocular accommodation. J Vis. (2022) 22:4. doi: 10.1167/jov.22.9.4, 35925580 PMC9363677

[32] Sweeney DF Millar TJ Raju SR. Tear film stability: a review. Exp Eye Res. (2013) 117:28–38. doi: 10.1016/j.exer.2013.08.010, 23973716

[33] Hazra D Yotsukura E Torii H Mori K Maruyama T Ogawa M . Relation between dry eye and myopia based on tear film breakup time, higher order aberration, choroidal thickness, and axial length. Sci Rep. (2022) 12:10891. doi: 10.1038/s41598-022-15023-x, 35764689 PMC9240066

[34] Szczotka-Flynn LB Maguire MG Ying GS Lin MC Bunya VY Dana R . Impact of dry eye on visual acuity and contrast sensitivity: dry eye assessment and management study. Optom Vis Sci. (2019) 96:387–96. doi: 10.1097/OPX.0000000000001387, 31116166 PMC6544497

[35] Xi L Qin J Bao Y. Assessment of tear film optical quality in a young short tear break-up time dry eye: case–control study. Medicine. (2019) 98:e17255. doi: 10.1097/MD.0000000000017255, 31577717 PMC6783147

[36] Liu H Thibos L Begley CG Bradley A. Measurement of the time course of optical quality and visual deterioration during tear break-up. Invest Ophthalmol Vis Sci. (2010) 51:3318–26. doi: 10.1167/iovs.09-4831, 20107168 PMC3990466

